# CT-guided Hookwire localization before video-assisted thoracoscopic surgery for solitary ground-glass opacity dominant pulmonary nodules: radiologic-pathologic analysis

**DOI:** 10.18632/oncotarget.22551

**Published:** 2017-11-20

**Authors:** Hao-Zhe Huang, Guang-Zhi Wang, Li-Chao Xu, Guo-Dong Li, Ying Wang, Yao-Hui Wang, Xin-Hong He, Wen-Tao Li

**Affiliations:** ^1^ Department of Interventional Radiology, Fudan University Shanghai Cancer Center, Xuhui, Shanghai 200032, People's Republic of China

**Keywords:** ground-glass opacities, pulmonary nodules, radiological feature, Hookwire localization, video-assisted thoracoscopic surgery

## Abstract

The optimal screening or treatment strategies of solitary pulmonary nodules especially ground-glass opacities (GGOs) remain controversial. With CT-guided Hookwire localization, it is accurate to find the small lesions during video-assisted thoracoscopic surgery (VATS). In this study, we evaluate the efficiency and safety of CT-guided Hookwire localization of GGO-dominant (GGO component > 50%) pulmonary nodules before VATS and investigate the correlation between the radiologic features and pathology. From April 2008 to April 2014, a total of 273 patients with solitary GGO-dominant pulmonary nodules were included. Tumor size was 12.4 ± 5.7 mm in diameter, including 208 pulmonary adenocarcinomas and 65 benign nodules. Dislodgement occurred in six patients (2.20%) during surgery. Postoperative complications included asymptomatic needle track hemorrhage (27.1%), minimal pneumothorax (5.9%) and hemoptysis (0.4%). In 208 (76.2%) pulmonary adenocarcinomas, 82 nodules showed ≥90% GGO and 126 showed 50%≤GGO<90%, while 84 nodules staged as T_1a_N0M0, 96 staged as T_1b_N_0_M_0,_ and 28 staged as T_1c_N_0_M_0_. The multivariable analysis demonstrated that 50%≤GGO<90% (HR=2.459, 95% CI: 1.246-4.853, *P*=0.010), speculation (HR=3.911, 95% CI: 1.966-7.663, *P*<0.001), lobulation (HR=4.582, 95% CI: 2.149-9.767, P<0.001) and vascular convergence (HR=4.096, 95% CI: 1.132-14.824, *P*=0.032) were the independent risk factors to identification of the malignant GGO-dominant pulmonary nodules. In conclusions,CT-guided Hookwire localizati for GGO-dominant pulmonary nodules before VATS is a safe and effective procedure for accurate diagnosis and resection of indeterminate solitary pulmonary nodules.

## INTRODUCTION

With the development and popularization of low-dose spiral computer tomography (CT), more and more solitary pulmonary nodules (SPNs) especially ground-glass opacities (GGOs) are becoming a much more frequent finding on thin-section CT. A SPN which can be either solid or subsolid in attenuation at thin-section CT is defined as a round opacity of up to 3 cm diameter surrounded by lung parenchyma [1]. Subsolid nodule includes pure GGO (pGGO) and part-solid GGO. Part-solid GGO can be classified into GGO-dominant nodules and solid-dominant nodules based on the proportion of GGO and solid components [2]. Recent studies have reported that the GGO components of part-solid nodules on CT usually correspond to pre-invasive components of pulmonary adenocarcinomas on pathology, while solid components frequently indicate invasive components, the ratio of GGO component was important for predicting the prognosis [3–6]. Nonetheless, there is no consensus regarding the division of part-solid GGO into two categories associated with prognosis, the GGO-dominant lesion was always defined as a tumor with GGO component ≥ 50% in most studies [7–9]. Although GGO-dominant tumors (even clinical-T1b tumors) have been identified as minimally invasive and with recent 5-year survival rates exceeding 90% [10], there are up to 20-40% lesions classified as malignant [11]. According to the current guidelines for management of pulmonary nodules detected on CT images (from the Fleischner Society 2017), GGOs < 6mm are not recommended either routine follow-up or biopsy [12]. But the optimal screening or treatment strategies of GGO-dominant lung nodules remain controversial in different regions [13].

As traditional approaches for diagnosing suspicious lung lesions, transbronchial biopsy or percutaneous needle biopsy are frequently less reliable for ruling out malignancy because of inadequate tissue sample or failure of biopsy with 51.2% diagnostic yield [14–17]. With minimally invasive, video-assisted thoracoscopic surgery (VATS) has been routinely used as a diagnostic and therapeutic approach to manage SPN [18]. The sublobar or wedge resections with VATS not only has the advantage of removing lung nodules while preserving pulmonary function, but also making a definitive diagnosis by intraoperative frozen section to guide resection strategy. Some studies showed that well-selected use of sublobar resection could yield comparable prognosis to lobar resection for peripheral small-sized non–small-cell lung cancer [19, 20]. Furthermore, the survival rate was no less than open surgery in patients with stage I disease [21]. However, it is the difficult to confirm the small nodules located at subpleural or far from the pleura in VATS procedure, which are visible on pre-operative CT scans. With CT-guided Hookwire localization before VATS, we can mark the location as accurately as possible for surgical resection. However, there currently is little evidence in these patients who are optimal candidates for sublobar resection. Therefore identification of malignant GGO-dominant nodules which is suitable for VATS is necessary. The purpose of this retrospective study was to investigate the correlation between the radiologic features and final pathology and evaluate the efficiency and safety of CT-guided Hookwire localization before VATS for solitary GGO-dominant pulmonary nodules.

## RESULTS

### Technical success

The procedure of CT-guided Hookwire localization before VATS was successfully performed in all 273 patients with solitary GGO-dominant pulmonary nodules (the baseline characteristics are summarized in Table 1). The mean longest diameter of nodules size was 12.4 ± 5.7 mm (range, 3.7-30.0). The duration of localization procedure was 8.6 ± 3.3 min (range, 4-23). Four puncture postures have been adopted including prone position (45.8%), supine position (28.9%), left (14.3%) and right (11.0%) lateral decubitus to select the optimal puncture path. The distance from the edge of nodules to the pleural surface was 11.3 ± 9.7 mm (range, 0.6-49.7). The insertion distance of Hookwire was 78.4 ± 15.8 mm (range, 44.3-134.2) with 147 through the nodules and 126 within 10 mm of the target lesions (Table 2). The times of CT scanning were 5.2±1.3, and the average of total dose-length product (DLP) in each patient were 231.5±98.7 mGy·cm.

**Table 1:** The clinical characteristics of patients (n=273)

Characteristic	N (%)
Age, years	53.9±10.6 (27-80)
≥70	18 (6.59)
<70	255 (93.41)
Gender	
Male	91 (33.33)
Female	182 (66.67)
Smoking history	
Yes	216 (79.12)
No	57 (20.88)
Tumor size	
>6mm	16 (5.86)
6-8mm	47 (17.22)
<8mm	210 (76.92)
Histologic types	
Benign	65 (23.81)
AAH	23 (8.42)
Inflammatory cell infiltration	19 (6.96)
Hyperplasia of fibrous tissue	15 (5.49)
Granulomas	5 (1.83)
Hamartomas	3 (1.10)
Malignant	208 (76.19)
AIS	130 (47.62)
MIA	23 (8.42)
IA	55 (20.15)
Increased serum tumor marker	
CEA	4 (1.47)
CYFRA 21-1	58 (21.25)

CEA: carcinoembryonic antigen; CYFRA 21-1: cytokeratin 19 fragments, AAH: atypical adenomatous hyperplasia; AIS: adenocarcinoma in situ; MIA: minimally invasive adenocarcinoma; IA: invasive adenocarcinoma.

**Table 2:** Characteristics of Hookwire localization

Characteristics	Value
Duration (min)	8.3±3.2 (2.5-23.0)
Insertion (mm)	78.4±15.8 (44.3-134.2)
Relation of Hookwire and GGOs	
Through the nodules	147 (53.8%)
Within surrounding 10 mm range	126 (46.2%)
Complications	
Needle track hemorrhage	74 (27.1%)
Minimal pneumothorax	16 (5.9%)
Hemoptysis	1 (0.4%)

GGO: ground glass opacity.

VATS was conducted in 272 patients, including 167 patients with wedge resection and 105 patients with lobectomy and lymphadenectomy. Dislodgement occurred in six patients (2.20%) during surgery. However, thoracoscopic resection was still performed based on the small hematoma left on the pleural surface enabled the nodule to be localized. The mean time of VATS procedure was 42.3±6.1 min (excluding ~20 min for frozen-section examination), and the average hospital stays of 4.3±1.8 days. VATS was converted to Muscle-Sparing thoracotomy in one patient (0.4%) because of strong pleural adhesion.

### Complications

The CT-guided Hookwire localization and VATS procedure was well tolerated in all patients, without any major complications or procedure-related mortality. No intra- or postoperative mortality or morbidity was recorded. The incidence of asymptomatic needle track hemorrhage was 27.1% (74/273), minimal pneumothorax (pleural surfaces were < 10 mm from chest wall) was 5.9% (16/273), and hemoptysis after localization was 0.4% (1/273) (Table 2). After antitussive and hemostatic measures, the lesion of that hemoptysis patient was resected successfully.

### Radiological and pathological analysis

The 273 solitary GGO-dominant pulmonary nodules included 65 (23.8%) benign lesions and 208 (76.2%) primary pulmonary adenocarcinomas, with 23 atypical adenomatous hyperplasia (AAH), 130 adenocarcinoma in situ (AIS), 23 minimally invasive adenocarcinoma (MIA) and 55 invasive adenocarcinoma (IA) (Table 1). The malignant rate of lesions with 50%-90% GGO component was higher than that of over 90% (126/149, 84.56% vs. 82/124, 66.13%, *P*=0.004). The malignant nodules with over 90% GGO were found in 63 AIS, 10 MIA and 9 IA, while the malignant nodules with 50%≤GGO<90% included 67 AIS, 13 MIA and 46 IA. Among 208 primary pulmonary adenocarcinomas, 84 nodules staged as T_1a_N_0_M_0_, 96 T_1b_N_0_M_0_and 28 T_1c_N_0_M_0_ (Table 3). Among 84 T_1a_N_0_M_0_patients, the lesions with ≥90% GGO was observed in 41 patients, and 50%-90% GGO was observed in 43 patients. Among 96 T_1b_N_0_M_0_ and 28 T_1c_N_0_M_0_patients, ≥90% GGO and 50%-90% GGO were observed in 36, 60; 5, 23 patients, respectively (Table 4). There were significantly statistical differences between malignant and benign GGOs in terms of nodular size, percentage of GGO component, margin, spiculation, lobulation, vascular convergence and cavity (Table 5). However, no significant differences were shown in age, gender, smoking history, tumor marker, pleural retraction and calcification (*P*>0.005). The multivariable analysis demonstrated that 50%≤GGO<90% (HR=2.459, 95% CI: 1.246-4.853, *P*=0.010), speculation (HR=3.911, 95% CI: 1.966-7.663, *P*<0.001), lobulation (HR=4.582, 95% CI: 2.149-9.767, P<0.001) and vascular convergence (HR=4.096, 95% CI: 1.132-14.824, *P*=0.032) were the independent risk factors. There were 48 patients with all three risk factors.

**Table 3:** GGO component and Stage of lung cancer in different pathologies (n)

Subject	AIS	MIA	IA	Total
GGO omponent				
GGO≥90%	63 (30.29)	10 (4.81)	9 (4.33)	82 (39.42)
50%≤GGO<90%	67 (32.21)	13 (6.25)	46 (22.12)	126 (60.58)
Stage				
T1aN0M0	72 (34.62)	7 (3.37)	5 (2.40)	84 (40.38)
T1bN0M0	55 (26.44)	12 (5.77)	29 (13.94)	96 (46.15)
T1cN0M0	3 (1.44)	4 (1.92)	21 (10.10)	28 (13.46)

AIS: adenocarcinoma in situ; MIA: minimally invasive adenocarcinoma; IA: invasive adenocarcinoma.

**Table 4:** Percentage of GGO component in different TNM stages of lung cancer (n)

TNM stage	GGO≥90%	50%≤GGO<90%	N
T1aN0M0	41 (19.71)	43 (20.67)	84
T1bN0M0	36 (17.31)	60 (28.85)	96
T1cN0M0	5 (2.40)	23 (11.06)	28
N	82	126	208

**Table 5:** The radiologic and clinicopathologic features of 273 GGOs

Characteristics	Malignant (%)	Benign (%)	Total	OR	95% CI	*P*
Age (years)	54.5±10.3	51.9±11.4		1.024	0.997~1.051	0.082
≥70	15 (7.21)	3 (4.62)	18			
<70	193 (92.79)	62 (95.38)	255			
Gender				1.345	0.754~2.399	0.316
Female	142 (68.27)	40 (61.54)	182			
Male	66 (31.73)	25 (38.46)	91			
Smoking history				0.994	0.969~1.019	0.616
Yes	42 (20.19)	15 (23.08)	57			
No	166 (79.81)	50 (76.92)	216			
CEA (ng/ml)	2.00±1.51	1.81±1.23		1.172	0.929 ~1.478	0.180
>5.2	3 (1.44)	1 (1.54)	4			
≤5.2	205 (98.56)	64 (98.46)	269			
CYFRA21-1 (ng/ml)	2.74±1.44	2.58±1.28		1.145	0.924 ~1.420	0.216
>3.3	46 (22.12)	12 (18.46)	58			
≤3.3	162 (77.88)	53 (81.54)	215			
Tumor location						0.395
RUL	84 (40.38)	28 (43.08)	112	1.800	0.71~4.564	0.216
RML	13 (6.25)	2 (3.08)	15	3.900	0.711~21.406	0.117
RLL	38 (18.27)	12 (18.46)	50	1.900	0.664~5.434	0.231
LUL	58 (27.88)	14 (21.54)	72	2.486	0.904~6.836	0.078
LLL	15 (7.21)	9 (13.85)	24			
Tumor size (mm)	12.89±5.71	10.70±4.92				0.047
>6mm	8 (3.85)	8 (12.31)	16	0.273	0.097~0.767	0.014
6-8mm	35 (16.83)	12 (18.46)	47	0.795	0.382~1.657	0.541
<8mm	165 (79.33)	45 (69.23)	210			
GGO component	()	()		0.356	0.200~0.636	<0.001
≥90%	82 (39.42)	42 (64.62)	124			
50%-90%	126 (60.58)	23 (35.38)	149			
Margin				0.344	0.167~0.708	0.004
Irregular	187 (89.9)	49 (75.38)	236			
Regular	21 (10.1)	16 (24.62)	37			
Spiculation				6.926	3.748~12.801	<0.001
Yes	157 (75.48)	20 (30.77)	177			
No	51 (24.52)	45 (69.23)	96			
Lobulation				8.418	4.291~16.513	<0.001
Yes	141 (67.79)	13 (20)	154			
No	67 (32.21)	52 (80)	119			
bubble-like lucency				4.018	1.819~8.873	<0.001
Yes	75 (36.06)	8 (12.31)	83			
No	133 (63.94)	57 (87.69)	190			
Calcification				0.302	0.060~1.536	0.149
Yes	3 (1.44)	3 (4.62)	6			
No	205 (98.56)	62 (95.38)	267			
Pleural retraction				1.213	0.640~2.299	0.555
Yes	59 (28.37)	16 (24.62)	75			
No	149 (71.63)	49 (75.38)	198			
Vascular convergence				12.656	3.843~41.685	<0.001
Yes	79 (37.98)	3 (4.62)	82			
No	129 (62.02)	62 (95.38)	191			

Malignant (n=208), Benign (n=65). CEA: serum carcinoembryonic antigen, CYFRA 21–1: cytokeratin 19 fragments; RUL= Right upper lobe, RML = Right middle lobe, RLL = Right lower lobe, LUL = Left upper lobe, LLL = Left lower lobe.

## DISCUSSION

The limiting factors for a successful thoracoscopic resection were not only the small size of the nodule and/or distance from the pleural surface [18], the percentage of GGO component would also influence operation success rate because of the difficulty in being palpated or identified when VATS resection regardless of the nodule size and/or distance from the pleural surface [22]. Therefore, precise localization was a prerequisite for effective resection, and various methods have been reported using dyestuff (mainly methylene blue dye), wire or microcoils, contrast agents [23], radiotracers [24] under ultrasound [25], CT or real-time CT fluoroscopy guidance [26]. Each of these localization methods has its own advantages and disadvantages. Methylene blue localization can be easily performed which has a high success rate and short localization procedure time. The major disadvantage is that the blue dye may rapidly diffuse into the surrounding lung parenchyma. It also has been reported that a failure rate of around 13% due to either an excess of liquid injected or an error in deep nodule localization. With regard to CT-guided localization with microcoils, no wire is left protruding extrathoracic, and it may decrease the pain and discomfort of patients. However, intraoperative fluoroscopy is necessary during the VATS procedure which increases radiation exposure. Injection of radionuclides also achieves a high success rate (96–100%); nevertheless, it seems to have most of the same problems as methylene blue injection. The disadvantages include increasing radiation exposure and no opportunity to inject again if a technical mistake occurs. The procedure of contrast medium including lipiodol location is similar to Hookwire localization. Lipiodol can be retained in the lung parenchyma for a long time, and the reported success rate is almost 100%. However, the contrast medium could also induce embolisms because it is water-insoluble. Ultrasound-guided localization could observe the nodules in real time with low cost and no invasion. However, the procedure is highly operator-dependent and limited by the presence of air in the lung when complete lung collapse is not feasible.

So far, the most commonly used pulmonary nodules localization technique is CT-guided Hookwire localization, which was first proposed by Mack [27] in 1992. Our results showed that the combination of CT-guided Hookwire localization and VATS was safe and efficient, though it took us some time to repeat CT scans for definite location of needle. However, we just spent about 8.3 min to locate a lesion which was faster than 14 min by Ichinose reported [20], and shortened the time of VATS within 42.3±6.1 min and hospital stay within 4.3±1.8 days which were faster than 75±12 min of VATS without preoperative Hookwire localization and 7±2 days of standard thoracotomy respectively [11]. The Hookwire can anchorage within the lesion and does not change position when the lung is partially collapsed for surgery, which do help thoracic surgeon exert a slight traction to facilitate resection contributing to less postoperative pain and earlier return to normal pulmonary function.

All of 273 GGO-dominant lesions were localized successfully and asymptomatic needle track hemorrhage having less obstacle on resection which was the most common complication with the rate of 27.1% (74/273), which was within the range of 0–35.3% reported in the literature [28–30]. The incidence of minimal pneumothorax (pleural surfaces were < 10 mm from chest wall) was 5.9% (16/273) which was lower than the range of 7.5–37.9% reported in the literature [11, 28, 30–32]. The hemorrhage we met mostly were opacities around the hooks or along the needle paths at CT requiring no intervention, and the pneumothorax were asymptomatic causing no reactions on patients. However, there was one patient got hemoptysis after localization. The amount of hemoptysis was bright red and less than 5 mL, but no other complication was shown on CT, maybe due to the terminal bronchiole and bronchial arterial injury. After antitussive and hemostatic measures, the lesion was resected successfully.

Hookwire dislodgement might occur on the occasion where patients’ deep and acute respiration to increase friction between the chest wall and Hookwire or surgeons’ tenting of the lung surface to facilitate resection [33]. Therefore, every patient was told to breathe quietly; attentions must be poured into light traction on the wire to tent the lung during wedge resection because of the loss of intraoperative reference. What's more, enough insertion depth of Hookwire (2 cm beyond lesions) also prevented from dislodgement happening in our study [29].

In this study, a total of 76.2% of the 273 GGO-dominant pulmonary lesions were malignant. Though AIS and MIA will not tend to spread to regional lymph nodes or to metastasize according to the IASLC/ATS/ERS classification, and the disease-free survival rate in these patients is nearly 100% respectively [2]. Those with invasive adenocarcinoma have more aggressive disease and poorer prognosis [34, 35]. Therefore it is essential to deal with these nodules timely.

At the same time, there were still 23.8% benign nodules with more than 50% GGO component on CT imaging to be distinguished from lung cancer. In order to obtain the key differential points of these GGOs, we compared patients’ clinical information and radiologic characteristics of benign and malignant lesions. In univariate analysis, lesions size, GGO component, pool defined margin, spiculation, lobulation, bubble-like lucency and vascular convergence were the most important discriminators for malignant GGO-dominant pulmonary nodules. As is known to all, lesions size is one of most important prognostic factors. The diameter of malignant GGOs was larger than benign ones (*P*=0.006). In addition, we divided the 208 early pulmonary adenocarcinomas into T_1a_N_0_M_0_, T_1b_N_0_M_0_ and T_1c_N_0_M_0_according to tumor size, and results demonstrated that lesions with over 90% GGO component were less than 50%-90% GGO component in all stages. However, in multivariate logistic regression only 50%≤GGO<90%, spiculation, lobulation and vascular convergence was significantly correlated with malignant characteristics. The malignant rate of lesions with 50%-90% GGO component was higher than that of over 90% (84.56% vs. 66.13%). As for the GGO-dominant early stage diseases, a better prognosis have been demonstrated and the more extensive the solid portions, the poorer the prognosis [36, 37]. What's more, some special imaging features including spiculation, lobulation and vascular convergence could contribute to differentiate malignant from benign GGOs. All interstitial fibrosis and most of the MIA nodules demonstrated a polygonal or irregular shape with obvious lobulation and/or spiculation, whereas coarse spiculation in the tumor margin may indicate a malignant transformation to invasive adenocarcinoma [38]. Lobulation is a common finding of lung MDCT and is more frequently seen in malignant than in benign pulmonary lesions. It was found in the present study that the rate of lobulation in malignant GGOs was significantly higher than in benign ones (24.5% vs. 19.0%). Among 208 malignant, 79 showed vascular convergence. A vascular convergence sign has a higher likelihood of being malignant, which shows as convergence of vessels to the tumor on CT imaging and mostly applies to the peripheral tumors. In our study, there were no significantly statistical differences in terms of age, gender, smoking history or tumor markers between two groups maybe with the reason that the stage of lung cancer was too early to show their characteristics to differ from benign lesions. With respect to the concentration of serum carcinoembryonic antigen (CEA) and cytokeratin 19 fragments (CYFRA 21–1) level, they were just slightly higher in malignant group because the expression of tumor markers was positive correlation with tumor stages and the latter had higher expression in pulmonary squamous carcinoma.

However, there were several limitations in our study. First, the number of sample was not large enough to cover all kinds of diseases demonstrated as GGO-dominant lesions at thin-section CT. If we enrolled much more patients, the data would be much more convincible. Second, this study is a single center, retrospective, and non-controlled study. The optimal screening or treatment strategies of GGO-dominant lesions should be further studied. And the efficiency and safety of CT-guided Hookwire localization before VATS compared with other location should be prospective designed and further discussed.

In conclusion, the preoperative CT-guided Hookwire localization could help VATS resection of surgically invisible and nonpalpable GGOs easier, return pulmonary function to normal earlier and shorten operation duration and hospital stays without serious associated complications. The GGO component 50%-90%, spiculation, lobulation and vascular convergence were the independent factors to identification of the malignant and benign GGO-dominant pulmonary nodules.

## MATERIALS AND METHODS

### Patients

**Figure 1:**
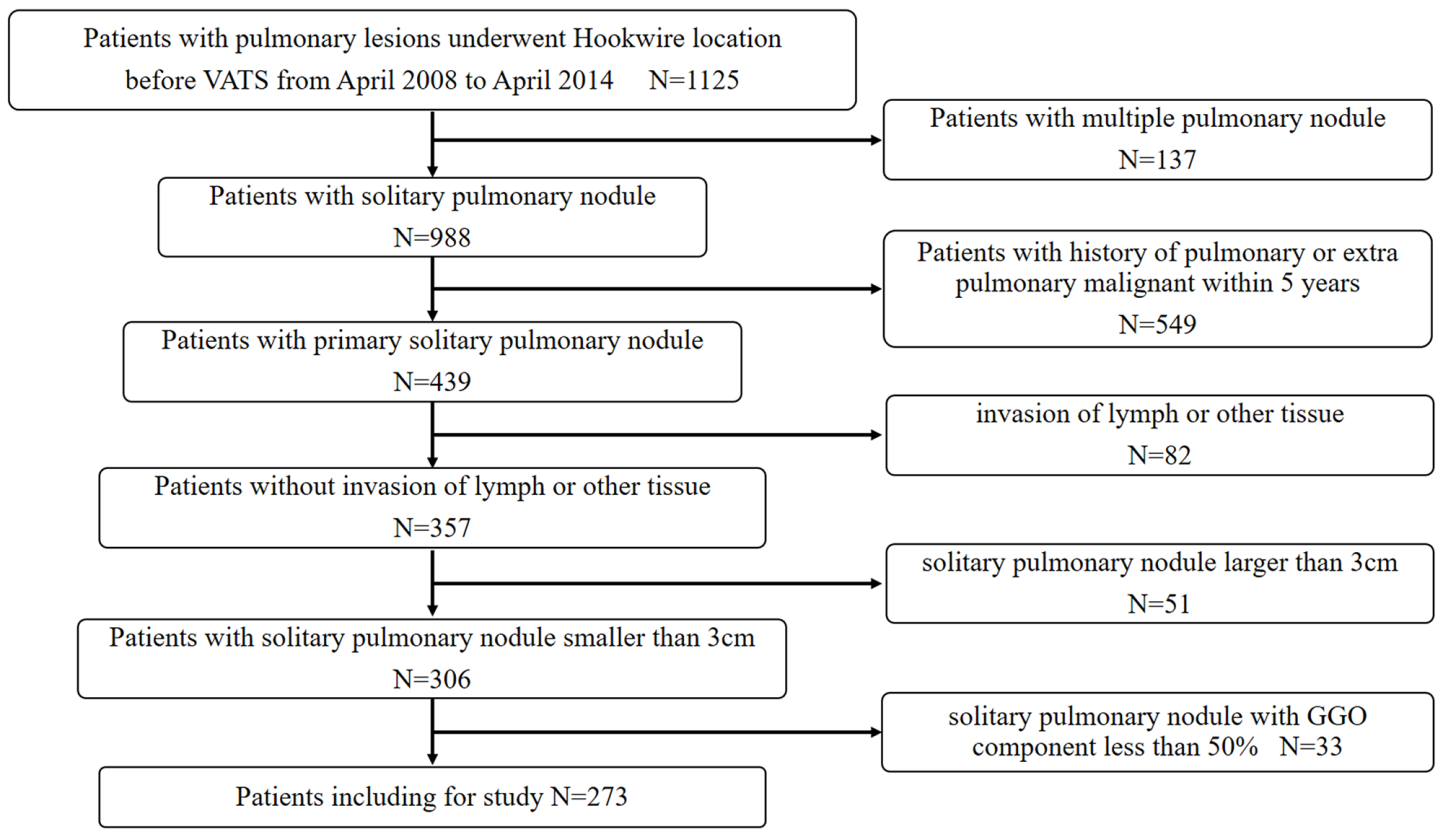
Flowchart of patient identification and inclusion

Institutional review board approval was obtained for this study, with waiver of informed consent for retrospective review of our clinical database. This retrospective study included patients with pulmonary nodules underwent CT-guided Hookwire localization before VATS in our center from April 2008 to April 2014. A total of 1125 consecutive patients initially were included in the study meeting the following inclusion criteria: (a) preoperative thin section CT images were acquired with our picture archiving and communication system; (b) available clinical data, including age, sex, smoking history and TNM stage; (c) a SPN with GGO; (d) 3 cm or smaller of the lesion size; (e) patients without invasion of lymph or other tissue. Among them, 371 patients were excluded due to the following exclusion criteria: (a) patients with multiple primary lung cancers, either synchronous or metachronous (n=137); (b) patients with pulmonary or extra pulmonary malignancies within 5 years (n=549); (c) patients with invasion of lymph or other (n=82); (d) solitary GGO larger than 3cm (n=51); (e) GGO component less than 50% (n=33). Finally, atotal of 273 patients (91 males, 182 females; mean age: 53.9±10.6 years, rang: 27-80 years) with solitary GGO-dominant lesions were included in the study. A flow chart of patient identification andselection is summarized in Figure 1.

### Thin-section CT evaluation

All patients performed preoperative thin-section CT. CT image acquisition was performed on a 64-detector row CT scanner (Brilliance 64 slice, Philips Medical Systems Inc., Cleveland, Ohio, USA) using unenhanced spiral acquisitions (tube voltage: 120 kVp; tube current: 130–200 mA; collimations: 0.625 mm; pitch: 0.758–1.015; rotation time: 0.5s; field of view: 350 mm; matrix: 512). Raw data of images were reconstructed at 0.67 mm thickness and 1 mm intervals using an edge-enhanced algorithm for lung parenchyma. All images were reviewed on a workstation, and radiologic characteristics were analyzed by post-processing software. Two thoracic radiologists (H.Z.H., G.D.L. with more than 8 years’ experience reading SPN studies) interpreted the CT images. The nodule size, attenuation value, location (upper/middle/lower lobe location or central/peripheral distribution), and morphological characteristics, including shape (classified as “regular” or “irregular”), margin (classified as “well defined” or “poorly defined”, lobulation or and speculation), internal structure (air bronchogram, bubble-like lucency and calcification), external structure (pleural retraction and vascular convergence) were assessed [39, 40]. They were blinded to the pathological diagnosis of the GGO nodules, and inconsistent interpretations of the CT findings were decided by consensus.

GGO is defined as hazy increased attenuation of the lung tissue without obscuring underlying vascular or bronchovascular structures in the lung window. The solid component was defined as an area of increased opacification that completely obscured the underlying vascular markings. All lesions were subsequently evaluated to estimate the extent of GGO with a thin-section CT scan 1- or 1.25-mm slice thickness. Measurements were performed with a window level of -500 to -700 Hounsfield units (HU) and a window width of 1500–2000 HU as the lung window. The percentage of GGO component was calculated as follows: [(D_GGO_– D_solid_)/ D_GGO_] × 100%, where D_GGO_ is the longest diameter of whole nodules including the GGO area and solid component on axial thin-section CT images, and D_solid_ is the longest diameter of the solid component [9, 37, 41].

### Hookwire localization under CT guidance

**Figure 2:**
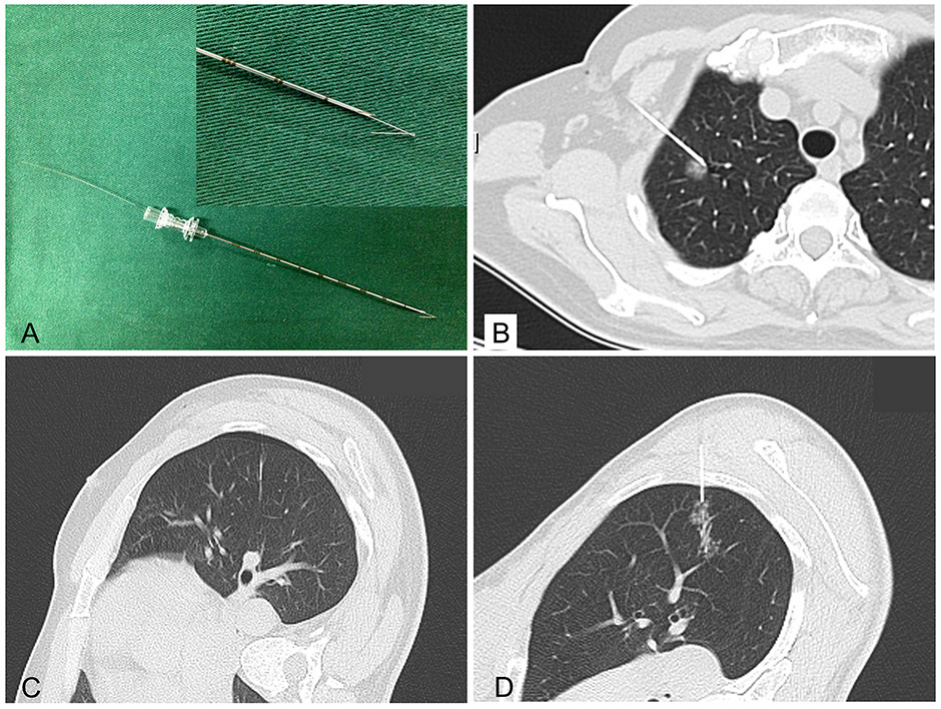
Hookwire system and the relative complications of localization **(A)** Hookwire system (Pajunk GmbH Medizintechnologie, Germany); **(B)** Hookwire has anchored the lesion; **(C&D)** Minimal pneumothorax and needle track hemorrhage.

Hookwire localization was performed on the day of surgery just before VATS. Patients were taken to the radiology unit and placed on the CT scan table in a prone, supine, or lateral decubitus position, depending on the location of the lesion. A calibrated cannula (21-gauge, 10 cm long) and a 20-cm long calibrated wire with a thorn constituted the Hookwire system (Pajunk GmbH Medizintechnologie, Geisingen, Germany; Figure 2A). The procedure were performed by one of four experienced chest interventional radiologists (X.H.H., G.D.L., L.C.X., Y.H.W.) who had 5–10 years of experience performing Hookwire localization. All procedure were performed under non-enhanced CT guidance with a low-dose technique (64×1.25 mm detector configuration, pitch of 1.4, table speed of 28 mm/rotation, 0.5 second gantry rotation, helical mode, 120 kVp localizing/100 kVp subsequent guiding scans with 40–60 mA) using a 64-detector–row CT scanner (SOMATOM Sensation 64, Siemens Medical Solutions, Forchheim, Germany). First, thorax CT scan (planning procedure) was performed to identify the location, size of the pulmonary lesion, as well as the relationship with the surrounding tissues. An optimal route was designed to traverse the shortest transpulmonary distance and avoid vital intrathoracic structures. After local anaesthesia, the cannula needle with Hookwire was inserted gradually through the chest wall into pulmonary parenchyma and placed as close as possible to the lesion (Figure 2B) under sequential CT guidance. Selected images were acquired in the area of interest with 3-mm-thick contiguous transverse CT sections (guiding procedure). Once optimal placement of the localizing needle tip was confirmed by CT, the outer cannula needle was withdrawn and the horn of Hookwire was placed adjacent to the target nodule. The patient was quietly breathing when the wire was placed. Repeated CT scan (control procedure) was performed to confirm whether the horn anchored the target lesion and evaluate associated complications including pneumothorax (Figure 2C), hemorrhage (Figure 2D) and so on. During the procedure, blood pressure, breathing rate and arterial oxygen saturation of patients were monitored. The Hookwire outside the chest wall was positioned carefully on the skin under sterile gauze. The patient was then transferred to operating room directly for VATS. In the meanwhile, images reconstructed by radiologists were uploaded to Picture Archiving and Communication System for thoracic surgeons to consult. Location time was defined as the duration time from first CT scan for identify the location to last CT scan for verify placement of the localizing Hookwire. Successful localization was defined as inserting Hookwire into the lesion or its surrounding 10mm range without obvious migration or dislodgement before VATS. The time of transferring to operating room was defined as time interval between CT unite and operating room.

### Video-assisted thoracoscopic resection

**Figure 3:**
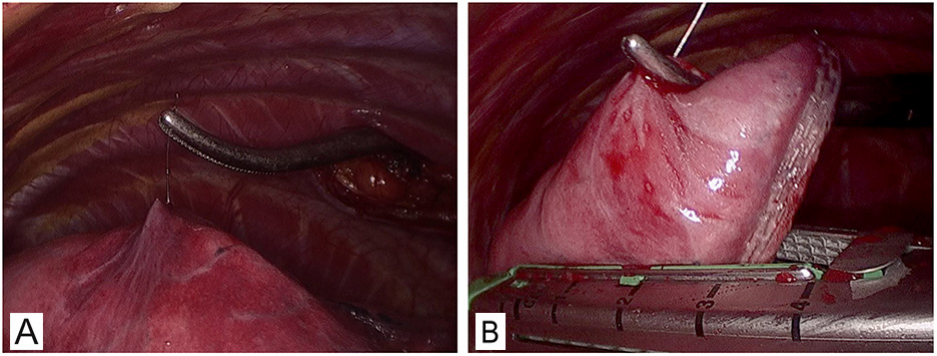
Resection of pGGO under VATS **(A)** A right lateral approach VATS view of the right upper lung. **(B)** The wedge resection directly guided by Hookwire with no obviously visible subpleural lesion.

VATS was performed under general anesthesia utilizing single lung ventilation via a double-lumen endobronchial tube. The procedure required two thoracic incisions of 10 mm, one for the thoracoscope at the intersection of posterior axillary line and the ninth intercostal space, the other one for the endoscopic stapler at the intersection of anterior axillary line and the seventh intercostal space. If necessary, a 5 mm thoracic port for the lung forceps between anterior and midaxillary line in the level of the third intercostal space would be required. During the procedure the Hookwire was raised and the lesion was sequentially resected completely (Figure 3). The resected wedge of lung tissue with Hookwire was packed into sterile gloves and withdrawn from the chest via an intercostal incision. If the hookwire had become inadvertently dislodged, the subtle subpleural hematoma that usually occurs at the entry site of the needle could be identified. After resection of the SPN, frozen-section examination was immediately performed on all specimens. If the pathological result was benign, a chest tube was inserted and surgery was completed after bleeding and air exclusion. If primary lung cancer was diagnosed by intraoperative frozen section, VATS lobectomy and lymphadenectomy were conducted. If metastatic tumor following wedge resection, the procedure was terminated before a multidisciplinary treatment scheme was established. In especially, certain problem including pleural adhesion or Hookwire dislodgement might result in converting VATS to an open thoracotomy. Successful surgery was defined as identification the lesions in the resected specimens with negative surgical margins by pathological examination after VATS. In addition, VATS had to be converted to thoracotomy on account of difficult localization, Hookwire dislodgement, bleeding or adhesions [11].

### Pathology diagnosis and histologic classification

After the tumors were removed by VATS, the specimen was sliced at the largest diameter of the tumor for sampling. The paraffin pathology diagnosis was made according to the newly revised classification of pulmonary adenocarcinoma by International Association for the Study of Lung Cancer (IASLC)/American Thoracic Society (ATS)/European Respiratory Society (ERS) [42]. If the lesions were diagnosed as adenocarcinoma, diagnosis was further stratified into AIS, MIA and IA. The predominant pattern was defined according to the histologic component with the greatest percentage. The TNM stages of tumor were according with the newest Eighth Edition of the TNM Classification for Lung Cancer [43].

### Statistical analysis

The duration of location, transferred to operating room, VATS procedure and hospital stay were recorded. The distance from the edge of nodules to the pleural surface and insertion section of Hookwire were measured. The difference of measurement data given as mean ± standard deviation was compared with the analysis of *t* test between two groups. Count data expressed in percentage was analyzed by the Chi-square test. The statistical analyses were conducted using SPSS 21.0 (SPSS Inc., Chicago, IL, USA). In univariate analysis, the differences of clinical pathologic factors between benign and malignant were analyzed with Pearson's chi-squared test or Fisher's test. A multivariate logistic regression analysis was used to identify the independent factors to predict malignant GGOs. *P* <.05 was considered statistically significant.

## Abbreviations

SPNs: solitary pulmonary nodules
GGOs: ground-glass opacities
VATS: video-assisted thoracoscopic surgery
AAH: atypical adenomatous hyperplasia
AIS: adenocarcinoma in situ
MIA: minimally invasive adenocarcinoma
IA: invasive adenocarcinoma
